# Multiscale and Extended Retrieval of Associative Memory Structures in a Cortical Model of Local-Global Inhibition Balance

**DOI:** 10.1523/ENEURO.0023-22.2022

**Published:** 2022-06-07

**Authors:** Thomas F. Burns, Tatsuya Haga (芳賀 達也), Tomoki Fukai (深井朋樹)

**Affiliations:** Neural Coding and Brain Computing Unit, Okinawa Institute of Science and Technology Graduate University, Okinawa 904-0495, Japan

## Abstract

Inhibitory neurons take on many forms and functions. How this diversity contributes to memory function is not completely known. Previous formal studies indicate inhibition differentiated by local and global connectivity in associative memory networks functions to rescale the level of retrieval of excitatory assemblies. However, such studies lack biological details such as a distinction between types of neurons (excitatory and inhibitory), unrealistic connection schemas, and nonsparse assemblies. In this study, we present a rate-based cortical model where neurons are distinguished (as excitatory, local inhibitory, or global inhibitory), connected more realistically, and where memory items correspond to sparse excitatory assemblies. We use this model to study how local-global inhibition balance can alter memory retrieval in associative memory structures, including naturalistic and artificial structures. Experimental studies have reported inhibitory neurons and their subtypes uniquely respond to specific stimuli and can form sophisticated, joint excitatory-inhibitory assemblies. Our model suggests such joint assemblies, as well as a distribution and rebalancing of overall inhibition between two inhibitory subpopulations, one connected to excitatory assemblies locally and the other connected globally, can quadruple the range of retrieval across related memories. We identify a possible functional role for local-global inhibitory balance to, in the context of choice or preference of relationships, permit and maintain a broader range of memory items when local inhibition is dominant and conversely consolidate and strengthen a smaller range of memory items when global inhibition is dominant. This model, while still theoretical, therefore highlights a potentially biologically-plausible and behaviorally-useful function of inhibitory diversity in memory.

## Significance Statement

Broadly, there are two types of neurons: excitatory and inhibitory. Inhibitory neurons are amazingly diverse compared with excitatory neurons. Why? Using a computational model with realistically-sized groups of excitatory neurons (representing memories) associated together in a network of memories, we highlight a potentially biologically-plausible and behaviorally-useful function of inhibitory neuron diversity in memory. Two findings in particular standout: (1) inhibitory diversity can quadruple the range of memory retrieval; and (2) balancing the strength of different inhibitory neurons’ influence on excitatory neurons can dramatically change how the network of memories become activated, balancing and extracting both geometric and topological information about the network.

## Introduction

The mechanisms by which our brains flexibly perform the complex tasks of learning and memory are not completely understood. Hebbian learning (Hebb, 1949), the relative increase in synaptic strength between neurons as a result of shared, causal activity, seems important. Hebb postulated memories were formulated in the brain by assemblies of highly-interconnected neurons (Hebb, 1949). Evidence for this “neuron assembly” hypothesis was found in hippocampus, where groups of neurons become synchronously activated in response to an animal’s spatial location, indicating a neural correspondence to and potential memory of the location (Harris et al., 2003). These memories are often mutually related, in physical or behavioral space for the case of navigation (Tolman, 1948), in reward space for the case of rewarded learning tasks (Dusek and Eichenbaum, 1997), in linguistic space for the case of language comprehension (Goldstein et al., 2021), and theoretically in any arbitrary semantic space for generalized graph-based reasoning (e.g., family trees; Whittington et al., 2020). How can the structure of these mutual relations be identified dynamically in cortical networks? Inhibitory mechanisms may hold an answer. Here, we computationally explore the possible role of inhibitory circuits in extracting graph-based relationships in the space of behaviorally relevant information.

The majority of experimental and computational work focusing on assemblies as representations of memory items has focused on the role of excitatory neurons. However, emerging evidence suggests inhibitory neurons play a nontrivial role in cortical networks. Throughout the brain, inhibitory neurons have classically been thought to coarsely keep excitation in check with a broad, nonspecific blanket of inhibition (Amit et al., 1994; Brunel, 2000). But more recent work has shown inhibitory neurons are tuned to specific external stimuli (Okun and Lampl, 2008; Xue et al., 2014), have specific associations with behavior (Dudok et al., 2021), have a large diversity of forms and functions within and across brain areas (Gouwens et al., 2020; Burns and Rajan, 2021), and form inhibitory assemblies (Zhang et al., 2017), often jointly with excitatory subnetworks (Otsuka and Kawaguchi, 2009; Koolschijn et al., 2019). A hallmark of many neuropathologies is inhibitory dysfunction (Amieva et al., 2004; Baroncelli et al., 2011; Burns and Rajan, 2022; Yao et al., 2022). If specific inhibitory dysfunction alone is sufficient for explaining these pathologies, then we could expect subtle inhibitory changes to cause dramatic changes in global function in complex tasks like those involving learning and memory. A greater understanding of the neurophysiological mechanisms underlying these changes may help us target treatments for such disorders and provide fundamental insight into the computational roles of inhibitory neurons in such circuits.

Previous modeling work in a formal model with binary neurons (Haga and Fukai, 2019) has shown how anti-Hebbian learning (i.e., involving inhibitory synapses) in an associative memory model was able to extend the span of association between mutually-related memory items organized in a simple ring structure, compared with a regular Hebbian learning rule (i.e., not involving inhibitory synapses). Later work extended this formal model to arbitrary graph structures (Haga and Fukai, 2021). These results suggest inhibition may play a nontrivial role in relational memory systems. However, these models lacked biological features, most prominently a lack of distinction between excitatory and inhibitory neuron populations, breaking Dale’s Law. Dale’s Law (sometimes also called Dale’s Principle or Dale’s Hypothesis), first appearing in Eccles et al. (1954), is the view that a neuron’s terminals do not transmit multiple, differently-acting chemical or electrical signals to postsynaptic targets, e.g., an excitatory neuron has the exclusive electrical effect of exciting postsynaptic targets and never inhibiting them. Another limitation of prior work is that the excitatory assemblies were also not nearly as sparse as those seen in biology and the neurons took on binary states. Nevertheless, the results indicate global functional changes can result from subtle inhibitory changes (Ferguson et al., 2013; Rich et al., 2017). This study proposes a more realistic connection scheme of distinct excitatory and inhibitory neurons to embed sparse cell assemblies which represent memory items mutually linked through arbitrary graph structures. Formulated in this way, the model allows us to confirm the previous suggestion that a balance between local inhibition and global inhibition on cell assemblies determines the scale and extent of memories retrieved in a neural network. We show this for various naturalistic and artificial associative memory structures, including as a potentially behaviorally-useful function to maintain a choice distribution given a juncture or decision point in physical or memory space. We find a balance between local and global inhibition allows control over the range of recall within arbitrary graph structures, as well as graph clustering effects which may be useful in navigation and memory tasks.

## Materials and Methods

### Model

In order to embed memories in the network, we generate binary patterns as vectors of length $N^{E}$, the number of excitatory neurons. Then, the weight $T_{ij}$ of connections between any pair of excitatory neurons i and j is defined using these patterns. First, we create p random binary patterns (of 0 and 1 s) of length $N^{E}$, ${\left\{\xi_{i}^{\mu }\right\}}_{i=1,2,...,N^{E}}^{\mu =1,2,...,p}$, with probabilities for 0 and 1 as $Prob\left[\xi_{i}^{\mu }=0\right]=1-f$ and $Prob\left[\xi_{i}^{\mu }=1\right]=f$, and where we call f the “sparseness” parameter of the memory patterns. This means neurons can belong to one or more memory patterns, and can be expected on average to belong to p⋅f memory patterns. Memories are then embedded using a modified extended association rule (Griniasty et al., 1993; Amit et al., 1994) designed to allow association between memory items in an arbitrary graph structure where vertices are the memory patterns and edges represent an association of two memory patterns.

(1) $$T_{\mathrm{ij}}=\sum_{\mu =1}^{p}\xi_{i}^{\mu }{\;\xi }_{j}^{\mu }+\sum_{\mu =1}^{p}\sum_{k\in K}^{p}\xi_{i}^{\mu }{\;\xi }_{j}^{k}$$

Specifically where K is the set of memory patterns neighboring (adjacent to, in the graph theoretic sense) pattern μ in the associative memory structure, M (an example of the memory patterns μ and K is illustrated in the example shown in Fig. 2*A*). Although all values of $T_{ij}$ are defined, not all are non-zero, in fact, many can be zero. This is because Equation 1 defines increases in $T_{ij}$ only when those units are assigned to the same memory pattern or neighboring patterns. We could interpret this functionally as neurons belonging to the same or neighboring patterns are connected with probability 1 and with probability 0 (or not connected) to all other excitatory neurons. An example of the functional consequence of this connectivity can be seen in Extended Data Figure 1-1.

**Figure 1.**
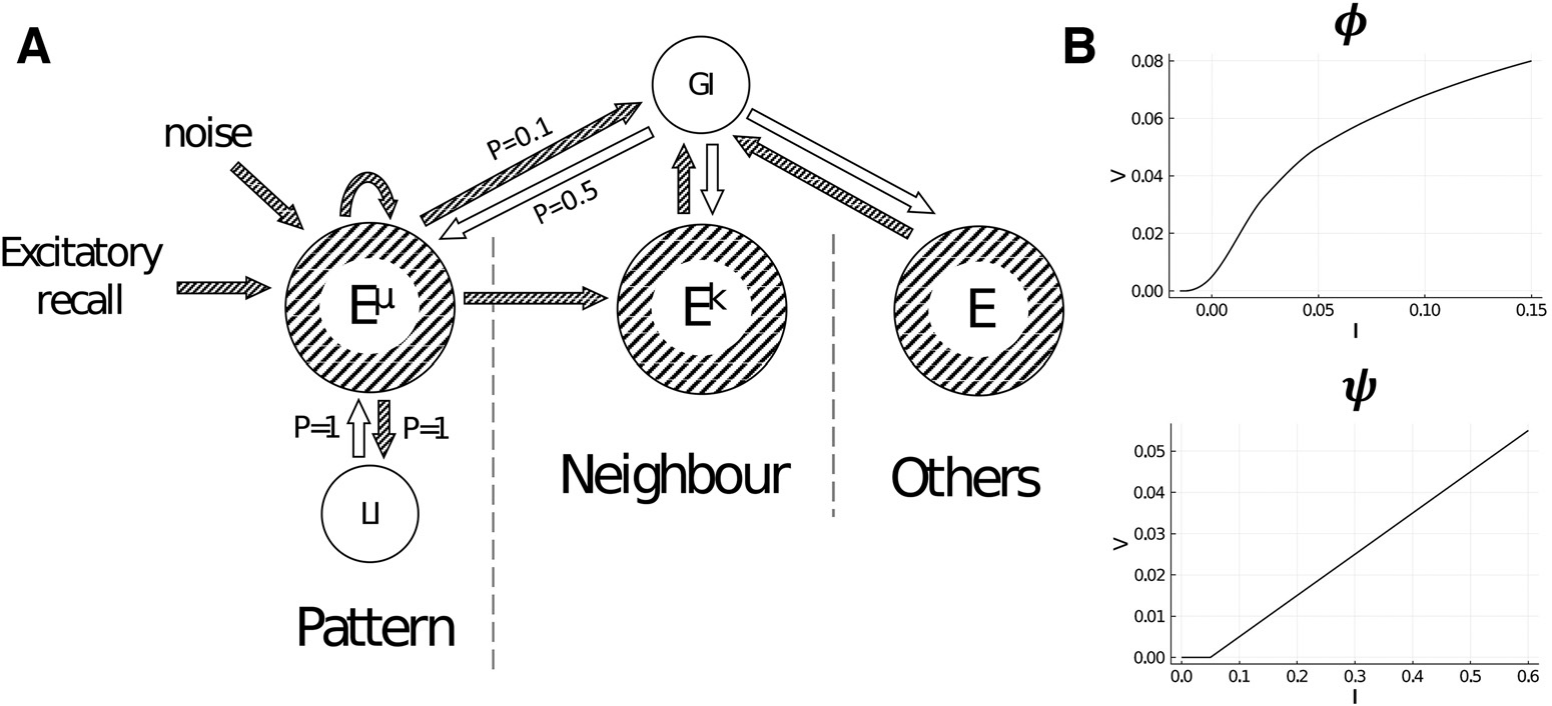
***A***, General schematic of the model from the perspective of a single memory pattern (E^μ^) and its connections to its respective local inhibitory population (LI), neighbors (E^k^), and the global inhibitory population (GI). Connection probabilities are indicated as values of P. To retrieve a pattern, excitation is given directly to a single pattern. Gaussian noise is also applied independently to all excitatory neurons. Key: striped/shaded arrows and circles indicate excitatory connections and populations, respectively, and unshaded arrows and circles indicate inhibitory connections and populations, respectively. N.B., although M consists of distinct memory patterns (and this distinction is necessary for the purposes of creating different associative memory structures), neurons can belong to any or all patterns (with independent probability of f to belong to any single memory pattern). Extended Data Figure 1-1 shows an example of the resultant activities over time for the stimulated pattern, neighbor, and other patterns. ***B***, Input-output functions for the excitatory neurons (ϕ) and inhibitory neurons (ψ; from Amit et al., 1994). For the excitatory input-output function, values of I above 0.15 are mapped to V=0.08, and the inhibitory input-output function continues linearly with the same slope for values of I above 0.6.

10.1523/ENEURO.0023-22.2022.f1-1 Extended Data Figure 1-1 An example of spiking rates of all units in the stimulated pattern, its first neighbor (pattern adjacent to the stimulated pattern), the neighbor’s neighbor (second neighbor), the neighbor’s neighbor’s neighbor (third neighbor), and all other patterns. This is from a simulation using c=0. Download Figure 1-1, EPS file.

Two populations of inhibitory neurons are also modelled, one with global connectivity (uniform connection probabilities as indicated in Fig. 1) of size $N^{G}$ and another with local connectivity, which is specific to each memory pattern, and has a total size of $N^{L}$, but where only $fN^{L}$ local inhibitory neurons participate in each pattern. Unless stated otherwise, we use $N^{E}=4,000$, $N^{G}=500$, $N^{L}=500$, and f=0.01, meaning that each pattern consists of a joint assembly of 40 excitatory neurons and 10 local inhibitory neurons. A general schematic of the model from the perspective of a single memory pattern is shown in Figure 1*A*.

**Figure 2.**
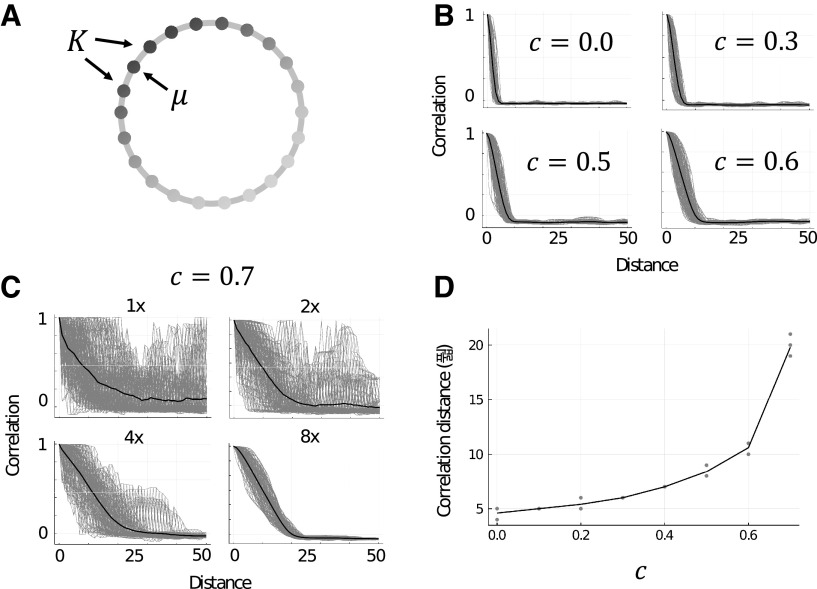
***A***, Illustration of M as a 1D chain. Arrows indicate the initially stimulated memory μ and the set of neighboring memory patterns, K, which are adjacent to μ in M. Shading of vertices indicates the strength of activity in each excitatory assembly (darker is more active). ***B***, Example trials showing the correlation of approximate steady-state activities of excitatory neurons with neighboring memories in a 1D chain associative memory structure. Gray lines are single trials (*n* = 100) and black lines are the mean of all trials. Panels show increasing the value of c to 0.6 (to a local inhibition dominant network configuration) approximately doubles the initial range of retrieval. ***C***, Example trials the same as ***B*** for c=0.7 with panels showing increasing sizes of networks (starting from 1x, which is $N^{E}=4,000$, $N^{G}=500$, $N^{L}=500$). This indicates a strong finite field effect which appears in the local inhibition dominant state. ***D***, Scatterplot showing the range of retrieval measure, D, increases with c. Gray dots are single trials (*n* = 5 per value of c) and the black line follows the mean of trials. Trials for c=0.7 were completed with $N^{E}=32,000$, $N^{G}=1,000$, $N^{L}=1,000$, and for all other values of c the trials were completed with the regular network size ($N^{E}=4,000$, $N^{G}=500$, $N^{L}=500$). Extended Data Figure 2-1 shows the stability of single units in these simulations of up to 5 s.

Neurons are modelled as proportions of their maximum firing rates, based on an established method (Amit et al., 1994; note: the following completely describes our implementation, including modifications, so readers need not be familiar with the prior work (Amit et al., 1994)). At each timestep, currents are calculated for each excitatory neuron $I_{i}^{E}$, global inhibitory neuron $I_{i}^{G}$, and local inhibitory neuron $I_{i}^{L}$:

(2) $$\tau^{E}\dot{I_{i}^{E}}=-I_{i}^{E}\;+\;\frac{1}{N^{E}\langle f\rangle }\underset{j\neq i}{\sum }T_{ij}V_{j}^{E}-\left(1-c\right)\frac{1}{N^{G}P^{EG}}\sum^{}J_{ij}^{GE}V_{j}^{G}-c\frac{1}{N^{L}f}\sum^{}J_{ij}^{LE}V_{j}^{L}\;+\;H_{i}^{ext}$$

(3) $$\tau^{I}\dot{I_{i}^{G}}=-I_{i}^{G}+\frac{1}{N^{E}fP^{GE}}\underset{i}{\sum }J_{ij}^{EG}V_{j}^{E}$$

(4) $$\tau^{I}\dot{I_{i}^{L}}=-I_{i}^{L}+\frac{1}{N^{L}f}\underset{i}{\sum }J_{ij}^{EL}V_{j}^{E}$$

and then converted into proportions of their maximum firing rates by:

(5) $$\dot{V_{i}^{E}}=\phi \left(I_{i}^{E}\right)+\varsigma_{i}$$

(6) $$\dot{V_{i}^{G}}=\psi \left(I_{i}^{G}\right)$$

(7) $$\dot{V_{i}^{L}}=\psi \left(I_{i}^{L}\right),$$

where $J_{ij}$ is the balanced connection weight between neurons i and j, $\tau^{E}=10ms$ and $\tau^{I}=2ms$ are the time decay constants, c∈[0,1] is the local-global inhibition balance, and $P^{EG}=0.5$ and $P^{GE}=0.1$ are the connection probabilities from excitatory to global inhibitory neurons and global inhibitory to excitatory neurons, respectively. The 〈f〉 term is the sum of expected firing rates based on the average degree of M, e.g., if M is a 1D chain, 〈f〉=f⋅1.5=0.015 (where by “1D chain,” we mean a set of vertices wherein each vertex is connected to exactly two other vertices in the set, such that they form a chain-link structure as illustrated in Fig. 2*A*). The 〈f〉 term therefore acts to normalize the excitatory-to-excitatory weights and does not affect the probability of neurons belonging to memory patterns. External input to the network is given by $H_{i}^{ext}=0.2$, the drive given to excitatory neurons in the pattern we wish to retrieve during the stimulation window, and $\varsigma_{i}\in N(0,0.0015^{2})$ is small Gaussian noise (independently drawn at every step, for every excitatory neuron). The input-output functions for the excitatory neurons (ϕ) and inhibitory neurons (ψ) are shown in Figure 1*B* and are from a previous study (Amit et al., 1994). The network’s forward dynamics (governed by Eqs. 2–7) are solved using the Euler method with step sizes of 0.1 ms.

10.1523/ENEURO.0023-22.2022.f2-1 Extended Data Figure 2-1 Each panel shows the spiking rates over 5 s for 100 random excitatory neurons drawn from separate, unique random seed simulations where M was a 1D-chain with p=100 and the regular protocol of stimulating one memory pattern for the first 80 ms was conducted. The six panels each used different values of c; however, because only a small random sample is drawn from the entire excitatory population in each case, no systematic differences are observable. Two aspects are common to all panels, however (1) within the first few hundred milliseconds, all neurons reach and then maintain an approximate steady state level of activity for the remainder of the simulation (despite jittering caused by external random noise); and (2) neurons can settle on many different mean spiking rates, and (proportionally) many remain quiet or have very low levels of spiking activity. Download Figure 2-1, EPS file.

The excitatory-to-excitatory weights are considered balanced by setting $T_{ij}=J_{ij}^{EE}$. We then balance the inhibitory-to-excitatory and excitatory-to-inhibitory weights based on $T_{ij}$. We balance the inhibitory-to-excitatory ($J_{ij}^{GE}$ and $J_{ij}^{LE}$) and inhibitory-to-excitatory ($J_{ij}^{EG}$ and $J_{ij}^{EL}$) connections by calculating the sum of each excitatory neuron’s presynaptic input in $J_{ij}$ and calculating the proportion of this sum compared with the mean sum of all excitatory neurons. This proportion becomes the connection weight, and obtains a mean of 1. In effect, this means excitatory neurons which receive stronger recurrent excitation than the mean excitatory neuron receive proportionally stronger local and global inhibition. Theoretically, this can be interpreted as a form of homeostatic normalization for the purpose of excitatory-inhibitory balance.

Associative memory structures M=(P,A) with |P|=p vertices (memory patterns) and edges (memory associations), A, is chosen and the model is instantiated according to the above procedure. We then choose a single pattern to receive external input to all of its excitatory neurons during the stimulation window, t=0ms to t=80ms, after which the network is left to settle into an approximate steady-state and stopped at t=500ms for analysis (we show representative examples in Extended Data Fig. 2-1 of simulations up to 5 s to demonstrate the stability of these approximate steady-states). The main variable of manipulation was the balance between local and global inhibition balance, c, where c=0 means only global inhibition is active, c=1 means only local inhibition is active, and c=0.5 means there is an equal contribution of both global and local inhibition in the network.

### Analysis

We noted changes to c systematically changed the number of memory patterns in M which became activated during the simulated memory retrieval phase (from t=80ms, when the external stimulation ended, to t=500ms, when the simulation ended), despite no change to the excitatory weights or structure of M. We refer to this phenomenon throughout this and following sections as an “extension” in the “range of retrieval” of the memory patterns. To quantify this extension in the range of retrieval given by changes in c, we tested M as a 1D chain with p=100. We stimulated each pattern and recorded the excitatory firing rates at t=480ms to t=500ms. With $W_{S}=20ms$ being the number of timesteps being averaged, we calculate the mean $\bar{V_{\mu }}$ and variance $\bar{\text{Δ}V_{\mu }^{2}}$ of the final firing rates for each memory μ by:

(8) $$\bar{V_{\mu }}=\frac{1}{W_{S}}\overset{N^{E}}{\underset{i=1}{\sum }}V_{i}^{\mu }$$

(9) $$\bar{\text{Δ}V_{\mu }^{2}}=\frac{1}{W_{S}}\overset{N^{E}}{\underset{i=1}{\sum }}{\left(V_{i}^{\mu }\right)}^{2}-\bar{V_{\mu }}.$$

The covariance between two memories μ and v is:

(10) $$COV_{\mu \;v}=\frac{1}{W_{S}}\overset{N^{E}}{\underset{i=1}{\sum }}V_{i}^{\mu }V_{i}^{v}-\bar{V_{\mu }}\bar{V_{v}}.$$

The correlation between two memories μ and v is:

(11) $$C_{\mu ,v}=\frac{COV_{\mu \;v}}{\sqrt{\bar{\text{Δ}V_{\mu }^{2}}\bar{\text{Δ}V_{v}^{2}}}}.$$

We then calculate the mean correlation between two memories at the shortest path distance d away from each other by:

(12) $$C_{d}=\frac{1}{p}\underset{\mu }{\sum }C_{\mu ,\mu +d}.$$

Finally, we quantify the range of retrieval D using the following algorithm:

1. Calculate $\left|C_{d-1}-C_{d}\right|$ for all d>1.

2. D is the first value of d for which the next Y memory patterns have $\left|C_{d-1}-C_{d}\right|<\varepsilon$. If no such D is found, $D=\frac{p}{2}$.

We use ε=0.05 and Y=5. Intuitively, this algorithm can be considered to estimate the distance in M from the initially stimulated memory pattern μ to the farthest sufficiently-active memory pattern d to quantify the range of retrieval.

**Figure 3.**
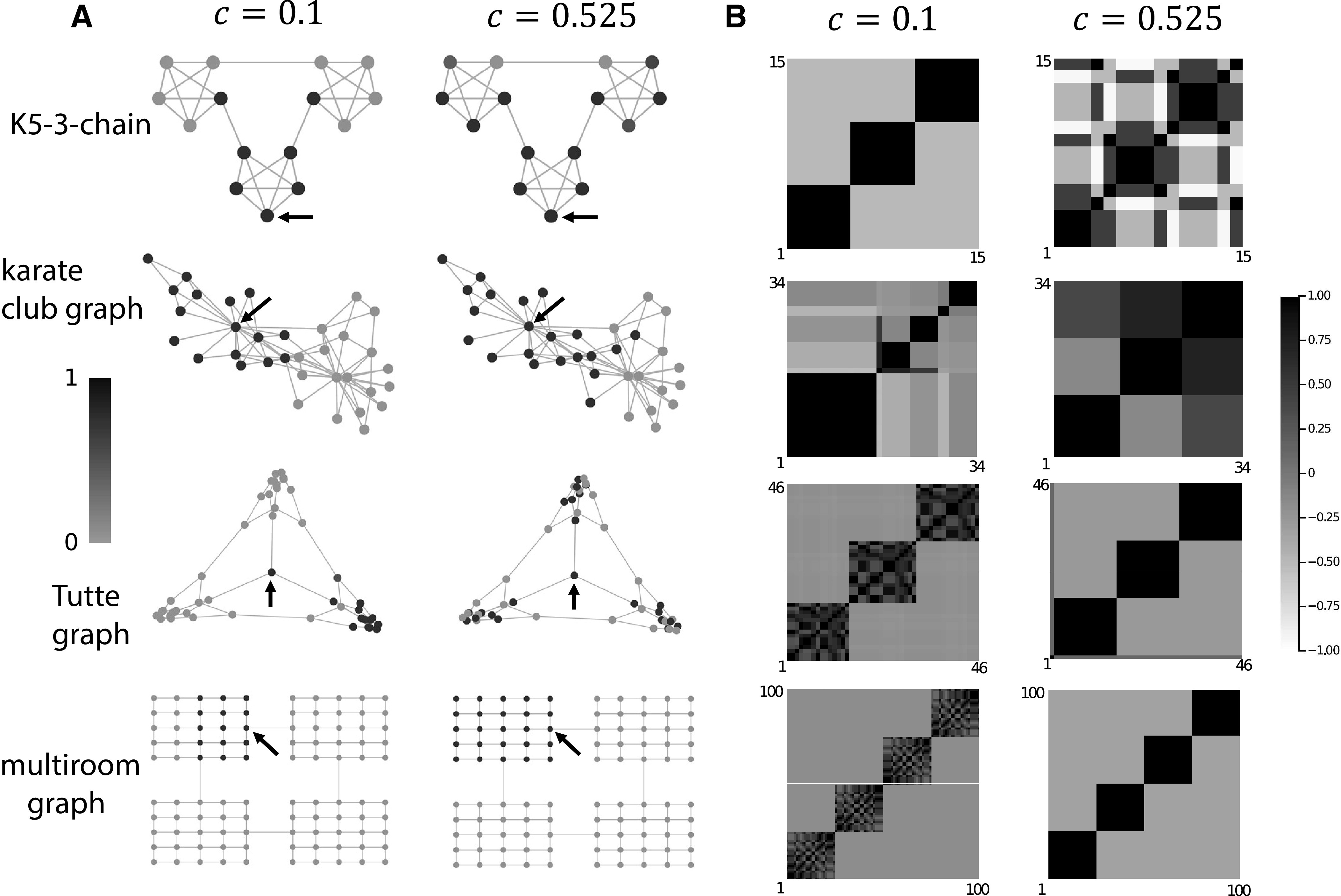
Each row of subplots in this figure corresponds to data from four different associative memory structures (graphs); from top to bottom: K5-3-chain, karate club graph, Tutte graph, and multiroom graph. ***A***, Example trials in the associative memory structures at two values of c. Vertices are shaded according to the sum of its neurons’ normalized activity (darker is more active). Arrows indicate the vertex which was stimulated at the beginning of the trial. ***B***, Correlations of approximate steady-state activities of excitatory neurons with all other vertices in the same associative memory structures and at the same two values of c as in panel ***A***. Vertices have been ordered such that those with similar correlations to other vertices are adjacent to illustrate the clustering effect that naturally arises from the network’s dynamics.

We observed how the activity of the excitatory population spread through associative memory structure for different values of c and across time. We chose to visualize this spread in three classical graphs, Zachary’s karate club graph (Zachary, 1977), the K5-3-chain (Schapiro et al., 2013), and the Tutte graph (Tutte, 1946), and one constructed graph representing a multi-room spatial environment which we call the multiroom graph. The karate club graph (Zachary, 1977) is a classical graph where each vertex represents a karate practitioner and edges connect individuals who interacted with each other outside of their karate training (Fig. 3*A*, second row). The K5-3-chain (Schapiro et al., 2013) is a set of three almost fully-connected graphs on five vertices, but where the edge between two vertices in each of the three almost fully-connected are cut and instead those vertices are connected to another almost fully-connected graph to form a single, connected graph (Fig. 3*A*, first row). The Tutte graph (Tutte, 1946; Fig. 3*A*, third row) has some notable graph-theoretic properties: (1) each vertex has exactly three neighboring vertices; (2) unlike many other graphs with the prior property [property (1)], there exists no Hamiltonian cycle in the Tutte graph, i.e., there is no path through the graph which visits each vertex only once, never uses an edge more than once, and returns to the original vertex it started at; and (3) there is a central vertex which connects to three separate “rooms” (clusters of vertices), despite there being a strong global symmetry in the number of each vertex’s neighbors [property (1)] while there is also a subtle asymmetry in the lack of a Hamiltonian cycle [property (2)]. We designed the multiroom graph to represent a spatial environment with four equally-sized rooms, each connected to two other rooms by centrally-located “doorways” on two sides of each room (Fig. 3*A*, fourth row). These graphs were chosen for their complexity, relation to or derivation from real-world analogues, and well-known graph theoretic features.

In order to quantify the similarity between the activity of the network and graph theoretic properties in the associative memory structures, we compared the approximate steady-state activity to the community detection and classification of vertices using the label propagation algorithm (Raghavan et al., 2007). We denote two vertices, e.g., μ and v, being members of the same community according to this algorithm with $LPA\left(v_{\mu },v_{v}\right)=1$ and $LPA\left(v_{\mu },v_{v}\right)=-1$ otherwise. Then, the clustering index for a given trial and its associated associative memory structure is given by the following:

(13) $$Q=\frac{1}{\left(p^{2}-p\right)}\underset{\mu }{\sum }\underset{\mu \neq v}{\sum }LPA\left(v_{\mu },v_{v}\right)C_{\mu ,v}.$$

The clustering index is a measure of how our model’s activity corresponds to topological features of M. To test how the activities correspond to geometric distance for arbitrary graphs, we define a local area around a vertex in M. This local area is the closed d-neighborhood of a vertex, i.e., the set of the vertex v and all vertices within distance d as measured by their shortest path to v. For a choice of d and v, we construct a local area function LA(μ|v,d) which assigns vertices in the local area with a value of 1 and −1 otherwise. We then calculate the geometric index by the following:

(14) $$R=\frac{1}{\left(p^{2}-p\right)}\underset{\mu }{\sum }\underset{\mu \neq v}{\sum }LA\left(\mu |v,d\right)C_{\mu ,v}.$$

### Code availability

The model was implemented using Julia 1.5.2. A copy of the code is publicly available at https://github.com/tfburns/BurnsHagaFukai (also see Extended Data 1).

10.1523/ENEURO.0023-22.2022.ed1 Extended Data 1 Code. Download Extended Data 1, ZIP file.

## Results

The general structure of the model is illustrated in Figure 1*A*. Memories are modelled as strongly-interconnected assemblies of excitatory neurons. Each memory item’s assembly is also interconnected to the assemblies of memory items which it is connected to in the associative memory structure, M. The associative memory structure can take on any form. Inhibition to the network is provided by two equally-sized populations: (1) a global inhibitory population, which has an excitatory to global inhibitory connection probability of 0.1 and global inhibitory to excitatory connection probability of 0.5; and (2) local inhibitory populations (one for each excitatory assembly), which are fully connected to individual excitatory assemblies in the associative memory structure. The balance between these two activities was governed by the parameter c: c→0 being strongly global, c→1 being strongly local, and c=0.5 being a balance between the two. A single trial is performed by giving a brief positive impulse (80ms) to a single excitatory assembly and then letting the network self-regulate its activity thereafter. This is similar to how a brief sensory stimulus of a single memory item can (even after the stimulus is removed) have persistent, representable activity and this activity can cause the retrieval of related memory items via cognition (Miyashita, 1988; MacDonald et al., 2011; Uitvlugt and Healey, 2019). We mostly analyze the approximate steady-state reached after 500ms.

### Extended range of retrieval

Setting M as a 1D chain with p=100 memory patterns, we simulated values of c from 0 to 1 in 0.1 steps. We found the range of retrieval extended gradually with increases to c (Fig. 2*A*). At c=0.7, the network showed a dramatic increase in noisy behavior, however this slowly subsided as we increased the size of the network, indicating a finite field effect (Fig. 2*B*). Compared with c=0, which had a range of retrieval of around 5, c=0.7 quadrupled this distance to 20 neighbors in distance along the 1D chain (Fig. 2*C*). In the range of c>0.7, we tested networks of sizes up to $N^{E}=128,000$, $N^{G}=4,000$, $N^{L}=4,000$ and found that in all cases the network activity was very noisy. Because of computational limitations, we did not test larger networks, however we speculate that sufficiently large networks are likely to exhibit even greater extensions to the range of retrieval but at smaller network scales are perturbed by noise from a finite field effect.

### Spread of excitation in associative memory structures

We also tested more sophisticated associative memory structures, namely: the K5-3-chain, karate club graph, Tutte graph, and multiroom graph. As in the 1D chain case, trials with values above c=0.7 often had noise, although the largest graph (multiroom) had stable trials with values of up to c=0.85. We also observed most graphs change in their excitatory activity most noticeably in the region of c=0.5 to c=0.6. We therefore chose to focus on two cases: (1) strong global inhibition (c=0.1), and (2) slightly stronger local inhibition (c=0.525; Fig. 3).

In most cases (karate club graph, K5-3-chain, and multiroom graph) excitation spread across a larger range of the associative memory structure when local inhibition was dominant than when global inhibition was dominant. The Tutte graph uniquely decreased the spread of excitation when activating its central vertex (Fig. 3*A*, third row, arrow). We suspect this is because of the unique topology of the Tutte graph and this central vertex—no other graph has strongly segmented “rooms” all neighboring a single vertex.

Correlations between the vertices (assemblies) of the underlying neurons (neurons belonging to those assemblies, see Eq. 11) showed different resolutions of clustering. For most graphs, there was a trend of more and small clusters at c=0.1 and then fewer, larger clusters at c=0.525. However, the K5-3-chain showed the breaking down of clusters and some strong negative correlations at c=0.525. We can see in Figure 3*B*, top row, that the graph is made up of pseudo-K5 subgraphs—groups of five vertices completely connected, except for two “boundary” vertices, which connect the pseudo-K5 subgraphs together. Within each pseudo-K5 subgraph, the three “core” vertices (those which are fully connected within the pseudo-K5 subgraph and not the boundary vertices) remain strongly correlated with one another while the two “boundary” vertices become almost equally correlated with their own pseudo-K5 subgraph and their neighboring subgraph and negatively correlated with the opposite subgraph. For the well-connected core vertices, c=0.525 also represents the level at which the spread of excitation almost covers the entire graph. This is quite unlike the other graphs tested. At c=0.1, in the karate club graph, approximately five clusters of strongly correlated vertices were present, whereas at c=0.525 this reduced to approximately three (Fig. 3*B*, second row). The Tutte and multiroom graphs showed a similar trend in consolidation of clusters at c=0.525 (Fig. 3*B*, third and fourth rows).

**Figure 4.**
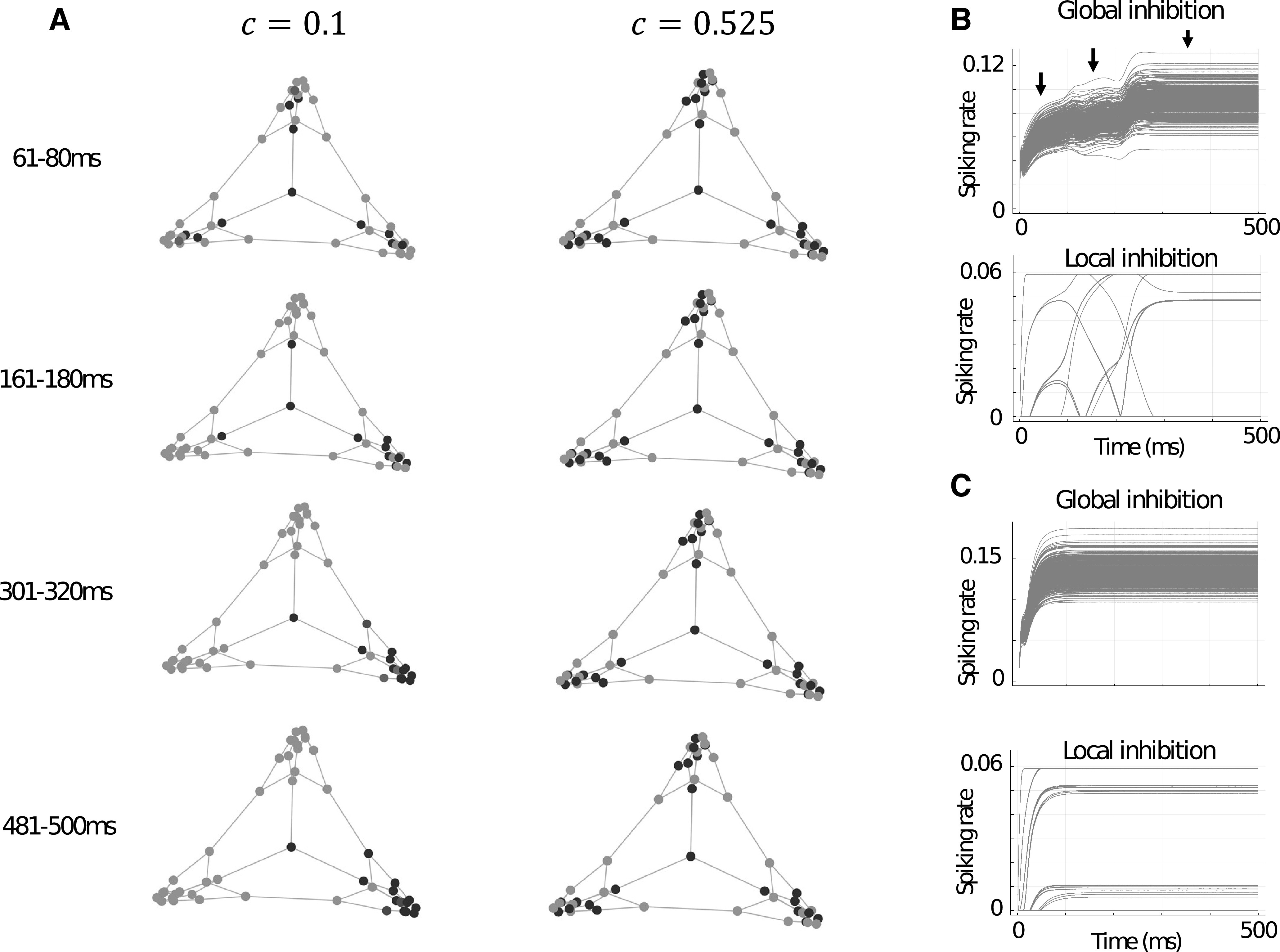
***A***, Example trials for memory item neuron activities in the Tutte graph during different time-windows for c=0.1 and c=0.525. The central vertex is activated for the first 80 ms of each trial. ***B***, Global and local inhibitory firing rates over time for the Tutte graph trial with c=0.1 shown in ***A***. Arrows illustrate three distinct modes or levels of global inhibition. ***C***, Same as ***B*** but for c=0.525.

We also observed how excitation spreads across the associative memory structure across time, after activation of vertices of interest, in the Tutte and multiroom graphs. For the Tutte graph we chose the central vertex, which branches off into three separate “rooms,” and for the multiroom graph we chose a location within one of the rooms that also led through a “doorway” to a neighboring room. We chose these vertices since they represent points of behavioral interest and ecological importance in animals—they are points at which an animal may make significant choice between which room to enter, explore, or exploit. In the Tutte graph, for c=0.1, there is initial activation of all three rooms (Fig. 4*A*). This is accompanied by a general rise in global inhibition and specific increases in the activity of local inhibitory populations connected to the respective active excitatory populations. However, at this early stage, one room is slightly more dominant in overall excitation (Fig. 4*A*, top-left panel, bottom-right “room”). This dominance appears to translate into gradual and then complete activity dominance compared with the other two rooms at the later time-windows. Contrastingly, for c=0.525, the activity of vertices in the Tutte graph is initially broader and this breadth of excitation is maintained steadily throughout the duration of the trial. We also see that the global and local inhibitory populations for c=0.525 (Fig. 4*C*) quickly stabilize in an approximate steady-state. In the case of c=0.1, the global inhibitory activity progresses through three distinct phases of activity (Fig. 4*B*, arrows): an initial rise, an unstable plateau, and finally a higher, stable plateau. Meanwhile, the local inhibitory activity for c=0.1 reflects the recruitment and release of various memory items before coming to an approximate steady-state at a similar time as the global inhibitory activity.

**Figure 5.**
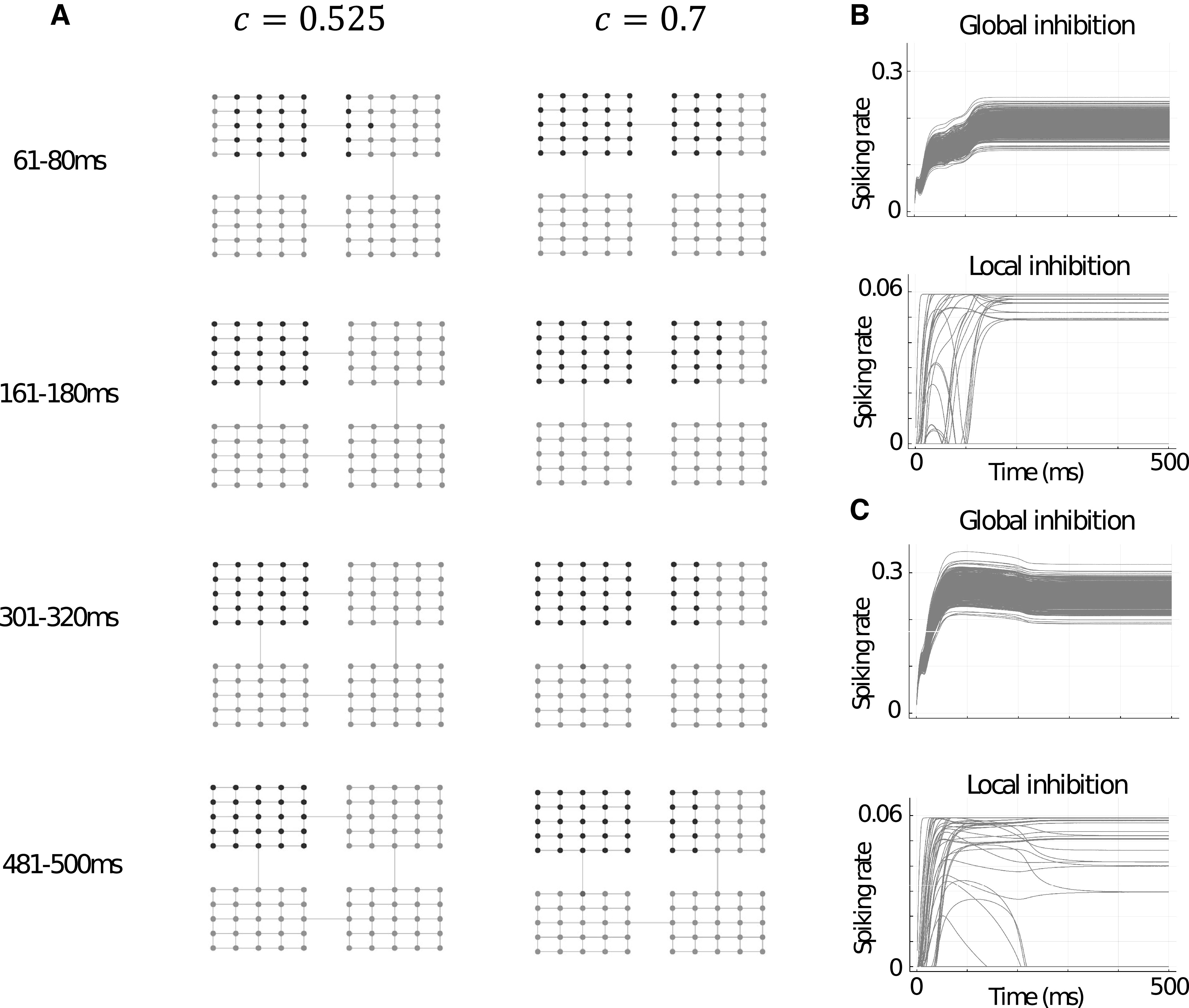
***A***, Example trials for memory item neuron activities in the multiroom graph during different time-windows for two different values global-local inhibitory balances, c=0.525 and c=0.7. A vertex beside to uppermost “doorway” is activated for the first 80 ms of each trial. ***B***, Global and local inhibitory firing rates over time for the multiroom graph trial with c=0.525 shown in ***A***. ***C***, Same as ***B*** but for c=0.7.

The multiroom graph showed a similar trend in broadening and maintaining a larger range of retrieval with increases in c. However, possibly because of the size of the network and because the chosen vertex was located within one of the rooms (thus biasing toward activation of that room’s other vertices, unlike the central vertex in the Tutte graph), observation of the effect required an increase in c. For illustration of the effect, we chose c=0.525 and c=0.7 (Fig. 5). Interestingly, in the case of c=0.7, initial broadening of the range of retrieval into the neighboring room (through the doorway adjacent to the memory item being stimulated) was slightly reduced and the first memory pattern of the room on the opposite doorway became active later in the trial.

The clustering and geometric indices, Q and R, for each graph, at different values of c are given in Table 1. Since R depends on a choice of distance d in the local area, we calculated R for all values of d (from 1 up to the diameter) and report the largest value of R (and its d) in Table 1 and for all values in Table 2. In general, the larger the value of Q, the more agreement between the community structure measured by label propagation and by the correlations of vertex activities in the final network states (by our model). High values of R indicate the final activity states are similar to geometric distance. We analyze the activity based on all neurons and a subset of neurons which reach a firing rate of at least 0.02 of the maximum firing rate during the simulation. We call this subset the selective neurons.

**Table 1:** Clustering and geometric indices for graphs at different values of c

		Clustering indices (Q)				Geometric indices (R)			
		All neurons		Selective neurons		All neurons		Selective neurons	
	Diameter	c=0.1	c=0.525	c=0.1	c=0.525	c=0.1	c=0.525	c=0.1	c=0.525
K5-3-chain	4	0.404	0.040	0.643	0.368	0.337 (d = 2)	0.393 (d = 4)	0.557 (d = 1)	0.592 (d = 2)
Karate club graph	5	0.098	−0.223	0.120	−0.266	0.111 (d = 2)	0.999 (d = 5)	0.191 (d = 4)	0.999 (d = 5)
Tutte graph	8	0.050	0.270	0.254	0.244	0.259 (d = 4)	0.389 (d = 4)	0.300 (d = 3)	0.464 (d = 3)
Multiroom graph	18	0.115	0.144	0.095	0.258	0.210 (d = 5)	0.375 (d = 5)	0.210 (d = 5)	0.375 (d = 5)

**Table 2:** Geometric indices (R**) for different graphs for all tested levels of** d

	K5-3-chain				Karate club graph				Tutte graph				Multiroom graph			
	c = 0.525		c = 0.1		c = 0.525		c = 0.1		c = 0.525		c = 0.1		c = 0.525		c = 0.1	
d	All	Selective	All	Selective	All	Selective	All	Selective	All	Selective	All	Selective	All	Selective	All	Selective
1	0.072	0.453	0.338	0.557	−0.722	−0.722	−0.029	−0.097	−0.019	0.137	0.029	0.092	0.073	0.073	0.052	0.052
2	0.334	0.592	0.338	0.443	0.223	0.223	0.111	0.133	0.178	0.325	0.174	0.230	0.170	0.170	0.122	0.122
3	0.379	0.296	0.234	0.129	0.711	0.711	0.018	0.105	0.341	0.464	0.258	0.300	0.270	0.270	0.178	0.178
4	0.393	−0.057	0.167	−0.071	0.971	0.971	0.087	0.191	0.389	0.459	0.259	0.277	0.345	0.345	0.207	0.207
5					1.000	1.000	0.081	0.190	0.302	0.287	0.193	0.181	0.375	0.375	0.210	0.210
6									0.204	0.102	0.122	0.078	0.360	0.360	0.196	0.196
7									0.151	0.002	0.084	0.023	0.314	0.314	0.170	0.170
8									0.140	−0.018	0.076	0.011	0.252	0.252	0.137	0.137
9													0.185	0.185	0.103	0.103
10													0.124	0.124	0.071	0.071
11													0.072	0.072	0.044	0.044
12													0.035	0.035	0.024	0.024
13													0.012	0.012	0.013	0.013
14													0.000	0.000	0.007	0.007
15													−0.005	−0.005	0.004	0.004
16													−0.008	−0.008	0.002	0.002
17													−0.009	−0.009	0.002	0.002
18													−0.009	−0.009	0.002	0.002
Weighted sum	3.448	2.297	2.383	1.543	10.743	10.743	1.000	2.195	7.827	5.935	5.089	4.185	14.626	14.626	8.996	8.996
Weighted mean	2.928	1.789	2.214	1.460	4.920	4.920	3.732	4.213	4.644	3.375	4.254	3.510	5.719	5.719	5.831	5.831
Weighted median	2	1	1	1	3	3	3	3	3	2	3	2	5	5	5	5

Clustering indices (Q) using only the selective neurons are generally larger than for all neurons, indicating these more-active neurons generally contribute positively to clustering. This is especially noticeable when the network settles into a state where assemblies take on a wide range of values (e.g., in the K5-3-chain graph for c=0.525). In general, the clustering indices indicate that given the size and topology of different graphs, different values of c have different propensities for clustering global characteristics.

Geometric indices (R) were generally greater than the clustering indices, indicating a greater emphasis of the geometry rather than the topology in these memory graphs at these values of c. Nonetheless, some topological information is captured and almost all of the geometric indices were of a comparable order as the clustering indices. As we increase c, the distance is increased or unchanged (i.e., not decreased). However, whether the clustering index increases with c depends on the structure of the graph. Importantly, either the clustering index or the distance becomes larger when c becomes larger, implying the approximate steady states can reflect the broader structure of the graph as the ratio of local inhibition to global inhibition is increased.

## Discussion

Previous modeling studies have conflated excitatory and inhibitory neuron identities and learning rules (Griniasty et al., 1993; Haga and Fukai, 2019) or ignored inhibitory neurons’ functional participation (Amit et al., 1994) in associative memory structure retrieval. This work uniquely disentangles excitatory and inhibitory neurons and uses sparse excitatory assemblies to demonstrate the potential functional role of global-local inhibitory balance in a more biologically-plausible setting. In the simplistic case of a 1D memory chain (like might correspond to discrete memories in a sequence of events through time), shifting inhibition to a locally-dominant state quadrupled the range of activation or retrieval. In the case of more sophisticated memory structures, globally-dominant inhibition tended to emphasize finer scale partitions of the memory structure and consolidated strong local associations. Whereas, locally-dominant inhibition tended to capture broader scale partitions and allow excitation to extend across a larger range of the memory structure.

It is important to emphasize these results are generated in the context of a memory structure which relies on the correlation of semantically close units, implying that memory retrieval in such a structure is functionally optimized when nearby units are correlated. Biological evidence for such correlations was first prominently shown in monkey anterior ventral temporal cortex by Miyashita (1988), which showed that the activity of units selective for arbitrary complex visual patterns was correlated by the stimulus-stimulus associations in the temporal ordering of the stimuli presentations. However, this kind of correlated, associative memory structure is not only found in the visual system, it is also noticeable and widely studied in hippocampus. Within a spatial environment, place cells representing nearby place fields show correlated activity (Monsalve-Mercado and Roudi, 2020) and can maintain correlations in the same environment over different tasks (Hampson et al., 1996), mostly because of overlapping place fields. When the environment changes, however, these correlations are typically inconsistent with one another (Alme, et al., 2014), suggesting contextual cues alter or switch between different memory structures.

In our study, we selectively stimulate single memory patterns and see memory retrieval of the pattern and surrounding associating patterns in ∼100–200 ms of simulation time. Is this biologically realistic? Single neurons in human medial temporal lobe which learn to selectively encode associative episodic memories within just a few trials can be recruited in subsequent activations within ∼500–700 ms (Ison et al., 2015); maximal pattern completion of cortical ensembles in visual cortex after subensemble optogenetic stimulation typically takes on the order of 2–4 s (Carrillo-Reid et al., 2019); biasing memory-guided spatial behavior by selectively stimulating clusters of place cells for ∼1 s has been shown to improve performance in reward-attaining behavior (Robinson et al., 2020). Therefore, the speed of memory retrieval in our model is likely on a faster timescale than should be generally expected in actual biological systems, although this could also be because of simplifications in the model, or disanalogous stimulation methods or assembly/memory structures.

Recent experimental evidence in mice (Rolotti et al., 2022) shows that when optogenetic techniques are used to induce place field formation in CA1 neurons, feedback inhibition limits the number of neurons which become activated, thereby limiting the size of the neural assembly which becomes activated by the induced place field. However, using disinhibition, this effect can be nullified and the neural assemblies can be made larger. Rolotti et al. (2022) showed such disinhibition can improve performance on a head-fixed spatial goal-oriented learning task via overrepresentation of the rewarded locations used for performance in the task. Another functional benefit of such disinhibition may be in rapid place field formation, as is seen in the behavioral timescale synaptic plasticity mechanism (Bittner et al., 2017; Zhao et al., 2020; Milstein et al., 2021). Our modeling results suggest similar effects may be possible without the use of disinhibition but rather simply via a rebalancing of the relative activity or strength between different inhibitory populations.

In Rolotti et al. (2022), the feedback inhibition comes from the hippocampus, but they do not explore distinctions between different inhibitory populations therein. There are many different types of inhibitory neurons, each with distinct connectivity, dynamics, and morphology (Pelkey et al., 2017; Burns and Rajan, 2021; Campagnola et al., 2022). In our model, we speculate that the “local” inhibitory neurons are parvalbumin-expressing while “global” inhibitory neurons are somatostatin-expressing, given there exists some evidence for such connectivity profiles in visual cortex (Adesnik et al., 2012; Litwin-Kumar et al., 2016). However, it is possible different areas may recruit and use inhibitory neurons and their circuits differently, for example to develop different scales of representations in hierarchical planning (Brunec and Momennejad, 2022). It could also be the case that there are even more functional groups of inhibitory neurons involved in these phenomena (e.g., see later in this discussion regarding a potential additional “global” inhibitory group for decreasing the correlation between neighboring memory patterns).

Inhibitory neurons also contribute to the initiation, maintenance, and modulation of rhythmic oscillations in local electrical activity (Traub et al., 1998; Fries, 2005; Bartos et al., 2007; Buzsáki and Wang, 2012; Aton et al., 2013). One example is in the pyramidal interneuron network gamma (PING) mechanism (Whittington et al., 1995), which can generate rhythmic dynamics which can ultimately result in the synchronous firing of excitatory neurons. Classically, the PING mechanism is thought of as involving just one group of excitatory neurons and one group of inhibitory neurons, and this is generally sufficient for the generation of PING dynamics. However, Rich et al. (2017) showed by expanding the diversity of inhibitory neurons into two groups with different recurrent disinhibitory connectivity, one weakly connected and one strongly connected, it is possible to achieve richer and more robust PING dynamics. Although we do not study disinhibition in our model and our techniques are substantially different to Rich et al. (2017), we partly followed in the theme of Rich et al. (2017; albeit in a different mechanism and showing a different phenomenon) by showing how by considering a greater diversity of inhibitory neurons acting simultaneously in a network, we are able to generate more interesting and novel dynamics. How inhibitory diversity related to different mechanisms or phenomena (e.g., the PING mechanism and the multiscale and extended retrieval of associative memory structures we demonstrate here) interact with one another is an open question for both computational and experimental neuroscientists.

Theoretically, in the absence of noise and with a sufficiently large network, an associative memory structure with N neurons can expect to accurately store (and retrieve via pattern completion) a maximum of 0.14⋅N memory patterns (Amit et al., 1985; McEliece et al., 1987), N/log(N) memory patterns if we permit more errors (Amit, 1992), and fewer if those patterns are correlated (Löwe, 1998; although exactly how fewer depends on the manner in which the patterns are correlated). In our case, the patterns themselves are not correlated, but rather they are created independently of one another and then correlated “spatially” in the larger memory structure M via excitatory weights between the memory patterns as described in Equation 1 and illustrated in Figure 1. Since we set the probability of neighboring memory patterns being connected to one another to be 1, the effective spatial correlation will also be 1. Past theoretical and numerical results (Cugliandolo and Tsodyks, 1994; Gandolfo et al., 1999) therefore indicate the memory capacity will be smaller than if the patterns were not correlated. However, if the spatial correlation is lowered, e.g., <0.5, the theoretical memory capacity can be the same as if there was no spatial correlation (for sufficiently large networks without noise) and the memory patterns will be sufficiently separated to allow accurate pattern completion.

Conceivably, it is possible to functionally enter into the range of spatial correlation <1 in our model without changing the connection probability between excitatory memory patterns and instead by sufficiently increasing the absolute strength of global inhibition while c=0. Such an increase in global inhibition will gradually suppress all patterns, with those most weakly activated dying out earlier. At the level of global inhibition just before all patterns are suppressed, one or more patterns will be minimally active, and this is likely less than the number of patterns active before the increase in global inhibition. However, increasing the strength of a global inhibitory population like shown in our model may not be biologically realistic, perhaps a more realistic scenario would be to recruit another global inhibitory pool, i.e., a second inhibitory neuron group which is globally connected to the excitatory population. However, this is beyond the scope of the current study and here we focus on the case of just one global inhibitory group and one local inhibitory group for each memory pattern. Nevertheless, for these reasons, the memory capacity of our model is less than the theoretical optimum because of the correlations between patterns, and as c→1 it becomes even less optimum since it is theoretically equivalent to increasing the strength of the correlations between neighboring memory patterns. A similar capacity effect is present in prior models with correlation between the memory patterns (Griniasty et al., 1993; Amit et al., 1994; Haga and Fukai, 2019), however this effect comes about by (in whole or in part) modifying excitatory weights whereas here we demonstrate this effect can be generated by modifications to inhibitory weights alone.

The effects generated by these modifications, such as stable extension in the range of retrieval, appears limited because of increases in noise in strongly local-inhibition dominant states. This is likely because of a finite field effect and may indicate a necessary minimum size of local excitatory-inhibitory assemblies for such states. For example, stability in the case of c=0.7 for the 1D chain case with sparsity of f=0.01 required a network size four times greater than the case of c=0 to maintain stability of retrieval, translating to excitatory assemblies of 160 neurons paired with 40 local inhibitory neurons. Although assemblies of ∼300 neurons have been used in optical microstimulation experiments in sensory cortex to drive behavior in mice (Huber et al., 2008), most recorded assemblies are on the order of tens of neurons (Harris et al., 2003; Fujisawa et al., 2008). Among other benefits, such sparsity is accompanied by theoretical energy efficiencies (Levy and Baxter, 1996) and in associative memory models can lead to fewer spurious memories (Hoffman, 2019). It therefore seems likely that for the described mechanism of local-global inhibition to have a stable functional effect in extending the range of retrieval, the presence of both local and global inhibition is required in finite, real-world networks with sparse assemblies.

Alternatively, it is possible this mechanism requires a hybrid sparse-dense coding schema, as has long been suggested operates in hippocampus (Barnes et al., 1990), cerebellum (Marr, 1969), and more recently in sensory areas (Laurent, 2002; Sakata and Harris, 2009). In such a schema, sparse assemblies report their activity to densely-connected assemblies which broadcast information to other sparse assemblies. In our model, we could consider the global inhibitory population as a densely-connected assembly which broadcasts the overall level of excitation in the network to all local, sparse assemblies. It is just not excitatory, as in classic dense-sparse schemas. Through this interpretation, a reduction in the relative strength of global inhibition (as in the unstable region of c>0.7) is equivalent to a gradual transition in the coding schema from sparse-dense to sparse. Thus, if the described local-global inhibition mechanism requires a sparse-dense coding schema, its instability when the coding scheme becomes sparse is expected. Associative memory structures which had more sophisticated topologies also showed unstable regions at high values of c, however less so when the graph was sufficiently large (such as in the multiroom graph). So, it is also possible this mechanism can be supported when the memory structure is adequately structured or large.

Extension of the range of retrieval was not simply the only apparent function of the inhibitory mechanism in sophisticated associative memory structures, the mechanism also permitted multiscale segmentation of the associative memory structure. Local-inhibition dominant states typically activated coarser topological segments of the graphs whereas global-inhibition dominant states consolidated activity in more densely associated clusters, highlighting finer topological features. These results were similar to those found in a more abstract model of binary neurons (Haga and Fukai, 2021), except that the current model was unable to eliminate the spread of excitation totally (as the more abstract model (Haga and Fukai, 2021) was capable of). This is because the current model does not include direct potentiation of excitatory weights, but rather modulation of local-global inhibitory balance. In this model, where association is embedded ubiquitously, to sustain highly-specific activity within a narrow range of memory items or even a single memory item, it is necessary to create very strong self-excitation within an assembly and have stronger overall inhibition with c→0. This demonstrates a general limitation that in a more biologically-realistic setting it may not be possible to fully eliminate or reduce association between items embedded in an excitatory memory structure through inhibitory modulation alone. Nevertheless, such inhibitory activity may cause dissociation through plasticity and learning mechanisms, as demonstrated in numerous psychological and biological studies (Anderson, 2003; Chiu and Egner, 2015; Schmitz et al., 2017; Anderson and Hulbert, 2021), which we have not investigated here.

An intriguing aspect of this inhibitory mechanism is its ability to dramatically affect not just the range of retrieval but also which parts of the memory structure become dominant given an initial stimulation. For example, it appears in global-inhibition dominant states, global inhibition drives a “winner-takes-all” dynamic (Grossberg, 1973) whereby only the globally strongest memories remain active. In local-inhibition dominant states, this “winner-takes-all” dynamic appears to dissipate and permit a general extension of retrieval, or a more egalitarian sharing of the winners. However, this extension can also be mediated and a “winner-takes-all” dynamic can appear at the peripheries of the retrieval range, with different peripheries competing against each other (Fig. 5*B*). This may be considered as a global state transition from “winner-takes-all” to “winner-shares-all” (Fukai and Tanaka, 1997). We therefore hypothesize an inhibitory mechanism like we have described may be used to aid in the learning or retrieval of graph-based cognitive tasks in cortical networks (Whittington et al., 2020; Wang et al., 2021). Cognitive control or exploitation of this mechanism might also occur in concert with, for example, gamma oscillations, which are strongly tied to inhibitory activity (Buzsáki and Wang, 2012). This may be especially useful when faced with competing behavioral choices and maintaining the distribution of these choices is meaningful, such as in perceptual decision-making (Najafi et al., 2020). Indeed, Roach et al. (2022) report that tuned local inhibition can alter the attractor dynamics of perceptual decision-making networks to balance between the speed or accuracy of perceptual decisions.

Probing such circuits and behaviors may provide insights on the potential influence such inhibitory mechanisms have on neuropathologies, especially those associated with cognitive defects (Amieva et al., 2004; Baroncelli et al., 2011). For instance, the coordination and interaction of inhibitory-driven oscillatory activity in hippocampus and prefrontal cortex is known to play a role in spatial memory tasks (Jones and Wilson, 2005) and spatial decision-making (Tavares and Tort, 2022). This coordination and interaction can be disrupted in epilepsy, leading to decreased behavioral flexibility (Kleen et al., 2011). Perhaps the associated behavioral deficits are in part because of maladaptations or dysfunction of local-global inhibitory balance or other subtle disruptions to networks involving multiple inhibitory neuron types.

While this study has made some advances over prior models (Griniasty et al., 1993; Amit et al., 1994; Haga and Fukai, 2019) in terms of improving the “biological realism” of the model, there exist many simplifications and unrealistic features in our model. We treat neurons as having a single point of intracellular space, i.e., without dendrites or specific morphology, which other than itself being unrealistic also prevents us from allowing different classes of inhibitory neurons to preferentially synapse onto different regions of other neurons, which is known to vary widely across inhibitory neurons (Otsuka and Kawaguchi, 2009; Burns and Rajan, 2021; Dudok et al., 2021). We also assume that joint excitatory-inhibitory assemblies are completely connected, which is a simplification that does not match biology (Otsuka and Kawaguchi, 2009; Koolschijn et al., 2019; Rolotti et al., 2022). Therefore, these and other limitations mean that whether and how actual biological networks achieve the same functional benefits we described here using inhibitory neuron diversity currently remains unknown. Experimentalists may therefore wish to design studies to test the presence or absence of such computational benefits in biological networks with diverse inhibitory populations.

In our model, making a seemingly subtle change to the network structure by introducing some of the complexities and diversities of inhibitory neurons had a profound impact on retrieval. We have shown how this phenomenon mainly persists in a sparse, associative memory structure which obeys Dale’s Law and has more biologically-plausible connections than prior models. We have also shown and discussed some of the potential functional roles of this mechanism in graph-based cognitive tasks and discussed how this mechanism may contribute to a type of sparse-dense coding schema.

## Synthesis

Reviewing Editor: Viji Santhakumar, University of California Riverside Decisions are customarily a result of the Reviewing Editor and the peer reviewers coming together and discussing their recommendations until a consensus is reached. When revisions are invited, a fact-based synthesis statement explaining their decision and outlining what is needed to prepare a revision will be listed below. The following reviewer(s) agreed to reveal their identity: NONE.

This manuscript address as important topic in the field of computational neuroscience, the role of various types of inhibitory interneurons in driving neural dynamics, as it pertains to memory retrieval of associative memory structures. The paper applies graph theoretical approaches to bridge a gap between past work on memory representations that used abstract models, and current experimental and modeling work that incorporates additional biological realism, such as neuronal cell types that follow Dale’s law, and have distance-dependent and cell-type-specific connectivity. The scope of the study, while narrow, is well-defined, and the analyses used are generally appropriate and clearly explained. The work is potentially of interest to both computational neuroscientists and experimental neuroscientists studying memory storage and retrieval. There are several concerns that need to be addressed so that this work is accessible to the broader interdisciplinary readership for which the findings will be of interest. Additionally, with the broader readership in mind, the manuscript could benefit from extended Discussion of the interpretation, implications, and broader consequences of their findings.

Specifically,

1. Both reviewers concur, and I agree, that the discussion could be improved for a neuroscience audience. Perhaps the implications and consequences of “extended retrieval range” could be related to ideas regarding pattern separation and pattern completion. Given that the connectivity of the memory structures are prescribed by the chosen set of input patterns, a chosen level of sparsity, and a chosen connectivity between memory patterns, there are a fixed number of clusters of discriminable patterns in each graph. However, as the dynamics of recurrent excitation and local/global inhibition evolve, the steady-state activities diverge from the initial activities and a lower number of clusters of discriminable patterns are produced. So the storage capacity of these memory structures is reduced by inhibition. Yet how the “extended retrieval range” constitutes a functional advantage of Dale’s law/functional diversity is not clarified.

Similarly better explanation of the motivation behind the computational analogues of memory retrieval studied here would be beneficial. For instance, the memory retrieval metric relies upon measuring the correlation of nearby units, implying that memory retrieval is optimized when nearby units are correlated with the stimulated memory. Is there biological evidence for this phenomenon, or is this an assumption used in the study of associative memory structures? Why are the time scales appropriate for memory retrieval, considering this would be memory at a time scale of less than a second? Is selectively stimulating the selected memory appropriate not just for the study of place-based memory, but memory generally? These issues should be considered in the discussion.

2. Several important details were not adequately reported in the methods and need to be addressed to improve clarity.

a. Were the neurons included in each memory pattern are unique: Figure 1 implies that they are, but the description of the model in the text is rather vague. It is not clear how the authors go about constructing their input patterns subject to both the sparsity constraint and the “degree of M” constraint? Please provide more detail.

b. The description of the “range of retrieval” metric is also quite confusing, exacerbated by the fact that the term “attractors” is not defined and the subscript t used on line 170 is also not defined.

c. The different graphs discussed in the paragraph beginning on line 173 should be explicitly detailed, and the multiple references to the “established method” of Amit et al needs to be explained within the text so that this work is approachable to those who are not familiar with this particular reference.

d. The authors should introduce Dale’s law for the broader readership (Line 73).

e. L96: What defines “neighboring”? Pairs of patterns with at least one shared active unit? Please provide some additional clarity in the manuscript. It could help to show examples of binary input pattern vectors that are neighboring and non-neighboring.

f. P5, L128, L154: At this point, it is not explained what is meant by a “1D chain”. It is clarified in Figure 2, perhaps a brief explanation and pointer to Figure 2 could be provided here in the Methods.

g. In Line 136 the synaptic weights are defined relative to the quantity T; this can be included directly in the equations presented just earlier for clarity.

3. The analysis of network dynamics between 480 and 500 ms as indicative of a “steady state” needs to be adequately substantiated by illustrating an example to show that that the steady state lasts longer than just 400 ms or by presenting the time course of the activity to justify the period that was selected for “steady state” analysis. Alternatively, longer duration simulations can be included to confirm that the behavior between 480-500 ms is indicative of a steady-state.

4. The move towards “biological realism” in the model is a definite strength and welcomed. However, some additional explanation of the extent to which the model can be considered realistic and the limitations may be worth emphasizing in the abstract, introduction and discussion for the benefit of the general neuroscience audience. For example, the choice of P=1 for the connection probability between a memory pattern and its local inhibitory population is a simplification that doesn’t match biology and may be misleading to more a more experimental audience.

5. A more extensive discussion of relevant computational studies examining the role of interneuron subpopulations would benefit the readers: two potential papers of interest may be “Dichotomous dynamics in EI networks with strongly and weakly intra-connected inhibitory neurons” and “Reduced inhibition in depression impairs stimulus processing in human cortical microcircuits”. Similarly, the idea that “global functional changes can result from subtle inhibitory changes” (Line 74) is something that has been the topic of contemporary computational study and thus this statement could benefit from some citations. Examples include “Experimentally constrained CA1 fast-firing parvalbumin-positive interneuron network models exhibit sharp transitions into coherent high frequency rhythms” and “Inhibitory network bistability explains increased interneuronal activity prior to seizure onset”.

Minor edits

1. Additional topics for discussion include

a. The difference between the dynamics in the Tutte graph relative to the other studied graphs could be probed/discussed further. For example, what aspect of this graph causes this difference?

b. Discussion of inhibitory disfunction as it pertains to neuropathologies in the introduction and relevance to diseases such as epilepsy, which is associated with cognitive defects could be beneficial.

2. Referring to curves in Figure 1B as “input-output functions” would be more appropriate than I?F curve which has specific experimental implication.

P8, L230: Caption for panel (D) is labeled (C).

3. P8, 9, Figure 3: The discussion of this figure would be easier to follow if the different graph types were labeled in the figure and figure legend, and discussed in the same order that they are presented in the figure.

4. P10: References to Figure 4 are mislabeled here as Figure 3.

5. P3, L93: There are numbered citations provided here, but the References are not numbered; “Memories are then embedded using a modified extended association rule [18, 29]”.

6. The supplemental data and tables should be made part of the main manuscript
